# Skewed Sociolinguistic Awareness of a Native Non-standard Dialect: Evidence from the Cypriot Greek Writing of Greek Cypriot Students

**DOI:** 10.3389/fpsyg.2017.02017

**Published:** 2017-11-22

**Authors:** Ioli Ayiomamitou, Androula Yiakoumetti

**Affiliations:** School of Education, Oxford Brookes University, Oxford, United Kingdom

**Keywords:** bidialectism, sociolinguistic awareness, written performance, non-standard varieties, language policy

## Abstract

Over the last 50 years, sociolinguistic research in settings in which a regional, social, or ethnic non-standard linguistic variety is used alongside the standard variety of the same language has steadily increased. The educational implications of the concomitant use of such varieties have also received a great deal of research attention. This study deals with regional linguistic variation and its implications for education by focusing on the Greek Cypriot educational context. This context is ideal for investigating the linguistic profiles of speakers of proximal varieties as the majority of Greek Cypriots are primarily educated in just one of their varieties: the standard educational variety. The aim of our study was to understand Greek Cypriot primary school pupils’ sociolinguistic awareness via examination of their written production in their home variety [Cypriot Greek (CG) dialect]. Our assumption was that, because written production is less spontaneous than speech, it better reflects pupils’ conscious awareness. Pupils were advised to produce texts that reflected their everyday language with family and friends (beyond school boundaries). As expected, students’ texts included an abundance of mesolectal features and the following were the ten most frequent: (1) palato-alveolar consonants, (2) future particle [en
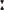
á] and conditional [ítan na] + subjunctive, (3) consonant devoicing, (4) CG-specific verb stems, (5) final [n] retention, (6) [én/ éni] instead of [íne], (7) CG-specific verb endings, (8) [én/é] instead of [ðen], (9) elision of intervocalic fricative [ɣ], and (10) CG-specific adverbs. Importantly, in addition to the expected mesolectal features that reflect contemporary CG, students included a significant and unexpected number of basilectal features and instances of hyperdialectism (that are not representative of today’s linguistic reality) which rendered their texts register-inappropriate. This led us to conclude that Greek Cypriot students have a skewed sociolinguistic awareness of variation within their first dialect and a distorted impression of their own everyday language. We argue that the portrayal of CG in its basilectal form was performed intentionally by students in an effort to distance themselves from a socially constructed identity of a rural, uneducated, and stigmatized non-standard-dialect speaker. The study is of international relevance as it deals with sociolinguistic issues that pertain to all bidialectal speakers.

## Introduction

Research in1pc2pc settings where regional, social, or ethnic linguistic varieties are used alongside a standard variety of the same language has burgeoned in recent years. Indeed, the issue of bidialectism is increasingly being viewed as a priority. Researchers have particularly aimed to identify whether the bilingual advantage, which has strong empirical support (Bialystok, 1988; Bialystok et al., 2012), extends to speakers of proximal dialectal varieties (Antoniou et al., 2014, 2016). In pursuit of this aim, a flourishing area of research has grown up around the premise that dialectal diversity may often have favorable outcomes and, in particular, that there is merit in assessing the potential for bidialectal programs in formal educational settings to produce beneficial learning outcomes. In Cyprus, Yiakoumetti (2006, 2007) demonstrated that the experimental introduction of bidialectal education (deploying the Cypriot Greek (CG) dialect alongside Standard Modern Greek) led to improved learning of the targeted standard variety. In Australia, Malcolm and Truscott (2012) provided evidence of positive influences on repertoire building when a bidialectal program (deploying Australian Aboriginal English alongside Standard Australian English) was introduced. Similarly, in Canada, improvement in Standard Canadian English reading skills was recorded when Canadian Aboriginal English was used alongside Standard Canadian English in bidialectal programs (Battisti et al., 2011; Ball and Bernhardt, 2012). In the Creole setting of Guinea-Bissau, Benson (1994, 2004) discovered that more students spoke in class and that there was less reliance on rote learning when bidialectal programs (deploying the native Crioulo alongside Standard Portuguese) were introduced. (For a review of studies on the outcomes from expanded use of Pidgins and Creoles in education, see Siegel, 2012.)

Research on bidialectal education has thus far focused exclusively on the effects of such education on educational linguistic varieties in bidialectal settings. In other words, bidialectal programs such as the ones just described targeted performance in students’ second variety, the educational standard. This is understandable considering that language policy goes hand in hand with power and prestige and that educational varieties are particularly prone to being associated with such value-laden concepts (Bourdieu, 1991; Spolsky, 2004).

This study focuses on the first dialect of speakers of proximal varieties. Specifically, it aims to explore bidialectal primary-school students’ sociolinguistic awareness as it is reflected in their written performance in their native non-standard regional dialect. The Greek Cypriot educational context served as vantage point for our exploration. This setting is ideal for investigation as it is representative of most bidialectal settings in which language policy disproportionately focuses on students’ educational standard variety. Traditionally, educational settings in which speakers employ the use of proximal varieties have been characterized by the anachronistic ideology that promotes exclusive use of an educational monolingual standard variety (Yiakoumetti, 2012). Both directly and indirectly, inclusion of varieties other than the prescribed standard is usually discouraged. In many cases, the very existence of these varieties is ignored and, in some cases, such varieties are openly banned from the classroom (Ndemanu, 2014).

Our research treatment was to encourage students to write in their dialectal native variety. The ultimate aim of this activity was to identify students’ sociolinguistic awareness via their written performance in a variety which can only be described as both (a) their most familiar variety but also (b) a variety which has never formed part of their formal education. In other words, students were asked to write in a variety which is their native variety but which they do not consider as being associated with formal writing or for formal use when writing in a school setting (Papapavlou and Pavlou, 2005). Our research facilitated an investigation of students’ written performance in the absence of any support (as current language policy comprehensively neglects students’ first dialect). Importantly, the project reflected students’ opinions as to what constitutes their first dialect and the policy’s effects on these opinions.

## Materials and Methods

### Setting of the Study

#### Greek Cypriot Sociolinguistic Landscape

Two linguistically related varieties are primarily used in Greek-speaking Cyprus: the CG dialect and Standard Modern Greek (SMG). (Similarly, Cypriot Turkish and Standard Turkish are used in Turkish-speaking Cyprus.) CG is the naturally acquired mother tongue of virtually all Greek Cypriots who go on to learn SMG via formal education. CG is widespread on the island as it represents the universal medium of everyday informal communication. SMG is the educational language variety.

Cypriot Greek is characterized by internal variability (Tsiplakou et al., 2016). Early descriptive studies (Contosopoulos, 1969; Menardos, 1969; Newton, 1972, 1983) presented CG as a geographical continuum which consisted of a set of basilects placed in opposition to a geographically defined acrolect, that of ελληνικά, Greek (Newton, 1972; Tsiplakou et al., 2006). Post-1974, these continuum varieties started to exhibit homogenization. This was primarily due to rapid demographic and social changes (as a result of the Turkish military occupation) and to heightened exposure to metropolitan SMG. Dialect leveling and koineization processes are still ongoing (Rowe and Grohmann, 2013). Today’s CG koine is almost entirely free of local variation as infrequent regional variants are fast becoming obsolete at phonological, morphosyntactic, and even lexical levels (Terkourafi, 2005; Tsiplakou, 2014). Some researchers argue that now CG can best be described in terms of a register or a stylistic continuum (rather than a geographically defined continuum) (Tsiplakou et al., 2016).

Contemporary CG is employed by all Greek Cypriots independently of their socioeconomic backgrounds. Various researchers on the island argue that today’s CG has expanded in domains which previously dismissed the dialect as inappropriate: its use has taken over both formal and informal domains replacing the use of SMG in a substantial number of cases (Themistocleous, 2009, 2010; Papapavlou, 2010, 2017). For example, contemporary CG (or at least its acrolectal levels) are now used in formal or semi-formal domains such as the court, public speeches, university lectures and the media. The dialect is indeed allocated an increasingly larger space in the current Cypriot mediaspace via the broadcasting of Cypriot sitcoms and telenovelas which are enjoying high popularity. We note that this recent expansion of CG is primarily associated with oral production. The emergence of CG in traditionally SMG domains has naturally granted the dialect more visibility and legitimization (Tsiplakou and Ioannidou, 2012).

Cypriot Greek speakers recognize a hierarchy of linguistic varieties which range from ‘heavily peasanty’ to SMG (Tsiplakou et al., 2006; Katsoyannou et al., 2006; Papapavlou and Sophocleous, 2009). (We further address this hierarchy in our Methods where we outline the various levels of language use along a continuum.) It must be emphasized here that the sociolinguistic and linguistic realities on the island offer its speakers a varied linguistic repertoire. Greek Cypriots have a wide range of features at their disposal. Their choices are therefore aligned to the context of the event of communication and may vary along the contemporary CG continuum.

In addition to the linguistic varieties already mentioned above (Cypriot Greek, Standard Modern Greek, Cypriot Turkish, and Standard Turkish), English is prominent in various domains such as the civil service and legal system. Western Armenian and Maronite Arabic are minority languages recognized within the European Charter for Regional or Minority Languages. Kurbetcha, a variety of Romani which is not well studied, is present but not recognized in the Charter (Hadjioannou et al., 2011).

In spite of linguistic diversity that is characteristic of Cyprus, the Greek Cypriot language policy treats SMG as the sole formal language of the national curriculum. The 2010 curriculum proved to be both an innovative and an abortive document. It was innovative for condoning the use of CG within formal education (Tsiplakou, 2015; Ministry of Education and Culture [MoEC], 2010). It was abortive in that its acknowledgment of CG led to heated debates which resulted in the rapid production of a replacement document which once again contained no reference to CG.

#### CG in Writing

Cypriot Greek is considered to be a spoken variety while SMG is the variety associated with writing. Apart from a number of improvised orthographic conventions (Chatziioannou, 1996; Yiangoullis, 2009; Katsoyannou et al., 2013; Coutsougera and Georgiou, 2014) that have been developed by poets, writers and lexicographers in an attempt to reflect unique dialectal sounds which do not exist in SMG (i.e., post-alveolar fricatives, post-alveolar affricates), the dialect is not codified and it does not have an established standard orthographical system.

Although rare, when writing occurs in the dialect, it is usually restricted to everyday informal communication events and involves forms of writing that are closer to speech such as instant messaging and online text-based communication among teenagers and young adults (Themistocleous, 2009, 2010; Sophocleous and Themistocleous, 2010). Due to the wide use of the Roman alphabet in online interactions, a romanized version of written CG (rather than one based on the Greek alphabet) is also very often employed, adding further to the multiplicity of writing systems that exist for the dialect. Research on the written form of the dialect has highlighted the repercussions of the lack of a unified way to represent the dialect and pointed out the need for its codification and standardization (Armosti et al., 2014; Papadima et al., 2014).

### Participants

One hundred and nineteen Greek Cypriot bidialectal students (63 boys and 56 girls) participated in the study. Students were in the fifth grade of primary education and all resided in the urban and semi-urban Limassol district. Their age range was 10–11 and all students’ native variety was CG. The participants formed a sociolinguistically homogeneous group as they were all born and raised in Limassol and all had Greek Cypriot parents. Students without Greek Cypriot parents and/or whose first variety was not CG were excluded from the analysis. In compliance with advice provided by the Cypriot Ministry of Education and Culture, we limited our sampled population to fifth graders as final-year sixth-grade pupils have additional demands associated with final exams. Our study was carried out in accordance with the recommendations of our institution’s Ethics Committee as well as those of the Cypriot Ministry of Education and Culture, with written informed consent from school headteachers and students’ parents or legal guardians. Access to information associated with students’ familial socioeconomic and educational profiles was not available so the influence of these factors on sociolinguistic awareness could not be considered.

### Primary Data-Collection Tool: CG Writing Task

Students’ sociolinguistic awareness was assessed via written texts which they were expected to produce in their native CG. Our assumption was that, because written production is less spontaneous than speech, it better reflects pupils’ conscious awareness. The task aimed to shed light on students’ ability to choose and produce the mesolectal register of contemporary Cypriot Greek.

During the design stages of the written task, we considered it essential for the language of the completed tasks to be characterized by non-test language (Luoma, 2004). We thus chose to develop a task that would simulate the usage of written CG in a real-life situation. To achieve this, a dialog between peers was chosen as the basis and the following scenario was presented to students for their responses.

Scenario: “Pambos and Koullis are two Cypriot pupils. Pambos lives with his family in Nicosia but they are soon relocating to Limassol. Pambos is apprehensive about this change and worries about feeling lonely in Limassol. He thus sends a message on MSN/Facebook or calls Koullis who resides in Limassol to share his worries.”

Instruction: “Imagine you are Koullis! Write the imaginary dialog you would have with Pambos. What do you think you would tell him to comfort him?”

It was thus inferred that the language of the tasks may be closely related to daily oral speech and it may also contain oral features that typically occur in online chat rooms or in telephone conversations. To assist students, explicit instructions regarding the linguistic variety they were expected to produce were provided. This guidance was as follows: (i) to write in the way they normally speak everyday outside of school with family members and friends and (ii) to use the Standard Greek alphabet to represent their pronunciation. In addition, the first three sentences of the script were provided as part of the task description such that students could continue on from these example sentences: “Γειά σου Κούλλη, ο Πάμποϛ είμαι! ΄Ιναμπου κάμνειϛ;;;; Éπιασα σε τηλέφωνο να σου πω κάτι… (Hello Koulli, it’s Pambos! How are you? I called you to tell you something ….)”. We note that the written guidance on how to conduct the task was provided in SMG to conform with usual classroom practice. However, as the request to write in the home variety was unusual, we also ensured that students were told orally what was requested of them. This was performed in CG.

The actual topic of the task ensured that the language of the text would reflect students’ everyday CG. While the instructions allowed for certain freedom to incorporate individual language choices, the scenario of the task clearly placed the target language event closer to mesolectal registers of CG (thus excluding language close to formal SMG but also excluding language close to basilectal CG). Despite the fact that compartmentalization of variants and registers is hazy (Tsiplakou et al., 2006), previous research has identified that Greek Cypriot speakers distinguish and recognize at least three levels of use (Sophocleous, 2006; Tsiplakou et al., 2006; Papapavlou and Sophocleous, 2009): (i) basilectal CG which corresponds to ‘heavy Cypriot, peasanty, βαρετά κυπριακά’, (ii) mesolectal CG which corresponds to ‘correct, tidied-up Cypriot, σωστά, σισταρισμένα κυπριακά’, and acrolectal CG which, despite approximating SMG, does not concide with it and perhaps corresponds to what has been named Cypriot Standard Greek (Arvaniti, 2010). If SMG were to be placed alongside the aforementioned levels, it would occupy the acrolectal end of the continuum (with the case of the language of school textbooks being a characteristic example). In light of this hierarchy, we note that formal SMG (primarily found in school textbooks), daily mesolectal CG (the form of language Greek Cypriots use in their daily lives), and basilectal CG (heavy Cypriot that includes features which are not part of Greek Cypriots’ active repertoire) clearly require the use of a distinct set of variants with which students are expected to be familiar.

In essence, our hypothesis was that the writing task would tend to encourage students to identify and correctly deploy (i) CG and not SMG and (ii) contemporary mesolectal CG and not basilectal CG. The students thus needed to resort to their repertoire and retrieve the unique structural features which constitute today’s mesolectal CG. This task may, at first glance, seem straightforward but the fact that contemporary mesolectal CG (which is employed by Cypriot Greeks on a daily basis) is almost exclusively associated with oral speech renders the task quite demanding. Indeed, students were often rather baffled when asked to write in their familiar home variety (D1) and this is a phenomenon which has also been observed previously by other researchers (Tsiplakou and Hadjioannou, 2010).

To conclude, the writing task aimed to elicit information on students’ perceptions about what is distinctively CG, thereby providing a richer insight into the nature of their register awareness.

### Secondary Data-Collection Tool: Interviews

Interviews were conducted to complement the writing task data. A subset of eight students, four boys and four girls, were randomly selected and interviewed individually. A semi-structured format was employed allowing for flexibility in the development of a casual, informal conversation. The issues covered in the interviews fell under two broad topics: (i) students’ perceptions of their two varieties and (ii) students’ views on their own language use.

### Data Codification and Analysis

The corpus of written scripts was scanned and imported into NVIVO 10 qualitative analysis software. The corpus was manually tagged for distinctive dialectal grammatical and lexical features as well as dialectal expressions as these formed the unit of analysis. Dialectal forms were identified according to the main and most marked characteristics of CG based on previous research. Specifically, to compose a list of features, both descriptive as well as empirical studies on CG that focus on individual CG phenomena were taken into account (Newton, 1972; Arvaniti, 1999; Pavlou and Papapavlou, 2004; Tsiplakou, 2004, 2006; Varella, 2006). In addition, three linguists who were native speakers of CG acted as independent raters and provided comments about the nature of a variety of features. This included assigning the features to the appropriate CG register (mesolect, acrolect, or basilect).

The resulting data were statistically analyzed via a general linear model approach to establish whether gender and/or class contributed significant effects. Response variables for (i) mesolectal, (ii) basilectal and (iii) hyperdialectal production were derived by weighting the number of instances per script over the total number of words in each script. As no significant effects were detected (*P* > 0.24 (1,115) for all potential predictors), these analyses are not reported. However, descriptive statistics including the mean number of mesolectal instances (±95% CI) and the percentages of scripts containing each of the three types of language use are provided.

## Results

Data from students’ CG texts highlighted two types of findings: (i) the expected mesolectal CG use and (ii) the unexpected non-mesolectal CG use. Both types are presented below.

### Mesolectal Features of Contemporary CG: 10 Most Common CG Items in Students’ D1 Writing

**FIGURE 1:**
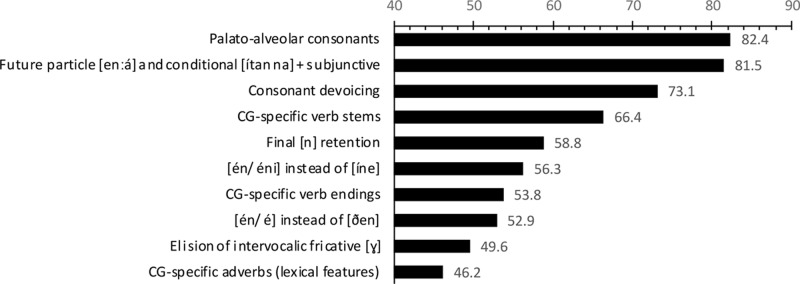
The 101pc most common mesolectal CG items as measured by percentage of student scripts which contained at least one instance of the item.

All 119 scripts contained mesolectal CG features. The mean number of mesolectal items per script was 27.8 ± 2.5 (95% CI) and the mean number of words per script was 96.4 ± 4.6 (95% CI). As each script is the discrete work product of a single and unique student, script is the appropriate sampling unit. The frequency of each item is thus presented as the percentage of scripts containing the item. The most common features are presented in the text below and in **Figure 1** (in order of frequency).

(1) Palato-alveolar consonants: In their attempt to render their writing as CG as possible, 98 out of the 119 students used /tʃ/, /ʃ/ and /Ʒ/ (palatal fricatives and affricates) 451 times. (Examples: ΄Ε**σσ**ει καμιά μ παράκα να τες βάλω μ έσα; [éʃi kamɲá mbaɾáka na tes válo mésa] is there any shed in which I can put them? Φέρε τες κóτες **τζ**ιαι τες τσoύρες μoυ. [féɾe tes kótes tʃe tes ts^h^
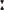
úɾes mu] bring.IMP my chickens and my goats)
(2) Future particle [en
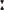
á] and conditional [ítan na] + subjunctive: the two morphological items were used by 97 students and occurred 386 times. (Examples: E**νά** μετακoμ ίω στη Λεμεσó [ená metakomío sti lemesó] I’m going to move to Limassol. **΄Hταν να σoυ πω** óτι **εννά** μετακoμήσω στη γητωνιά σoυ.[ítan na su po óti en
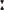
á metakomíso sti ɣitoɲ
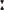
á su] I wanted to tell you that I’ll be moving to your neighborhood)
(3) Consonant devoicing: 87 students produced 202 instances of sequences of obstruents that followed the CG phonological process of voice assimilation. (During this process, the first voiced consonant changes into a voiceless consonant to assimilate with the adjacent voiceless /k/ sound.) (Example: παιχνι**θκ**ια [pexníθca] games)
(4) CG-specific verb stems: 79 students produced a total of 159 such instances which involved verbs that differed morphologically from SMG. (Example: **κάμν**ετε [kámnete] doing.2PL)
(5) Final [n] retention: the tendency to add a [n] sound at the end of a number of words was another strong CG phonological indicator. 70 students used this feature in 204 instances. (Example: την παρασκευή**ν** [tin paraskevín] on Friday)
(6) [én/ éni] instead of [íne]: The use of the CG form [én/éni] to express the 3rd person singular of the copula verb [íne]. This item was used by 67 students and occurred 150 times in total. (Example: **εν** τóσo ωραία [en tóso oréa] it’s so good)
(7) Cypriot Greek-specific verb endings: This type of feature appeared in the scripts of 64 students and occurred 117 times in total. (Example: πά**ει**ς σχoλείo [páis sxolío] go.2SG to school)
(8) [én/ é] instead of [ðen]: the form [én/ é] was found to be used in the place of the SMG [ðen] to express negation. It occurred in 63 students’ scripts 117 times. (Example: **εν** πάω μóνoν άμαν είμαι άρρωστη [en páo mónon áman íme árosti] I don’t go only when I’m unwell)
(9) Elision of intervocalic fricative [ɣ]: such instances were encountered in the scripts of 59 students 152 times. (Example: σί(γ)oυρα [sí(ɣ)ura] surely)
(10) Cypriot Greek-specific adverbs (lexical features). 55 students used adverbs that are specific to the dialect 95 times in their scripts. (Example: **δαμέ** [ðamé] here)

As can be seen from the list above of the ten most frequently used mesolectal CG features, the vast majority of the items found in students’ CG scripts are morphological and phonological. No syntactic features were found among the 10 most recurrently used items, while only one type of lexical item was recorded.

### Non-mesolectal CG Use

(1) Basilectal use: 55.5% of scripts included at least one basilectal CG instance. Items found under this category were dated or obsolete and not representative of contemporary CG. Some are restricted to isolated rural areas and others are almost entirely extinct. (Examples: σκoλείo [skolío] school, χέλω [çélo] want.1s, εγιώ [eʝió] I, γρóνια [ɣɾóɲ
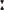
a] years, ευκαριστóσε [efkaɾistóse] thank you, τσίρης [ts^h^
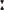
íɾis] father, ρα [ɾa] (form of address for female), πoά [poá] here).
(2) Hyperdialectism: 15.1% of scripts included at least one hyperdialectal CG instance. Hyperdialectisms were only ever present in scripts which also contained basilectal instances. Students showed a propensity to construct regional or pseudo-regional words mostly in terms of morphophonology by over-applying, re-introducing, and mis-adapting obsolete phonological and morphological features. Students’ hyperdialectism does not constitute part of contemporary CG or older stages of CG. (Examples: τηλεχωνo [tilexono] telephone.1s, τoυς γικoύς μoυ φίλoυς [tus ʝikús mu fílus] my own friends, ζωoλoγικóς τζίπoς [z
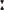
ooloʝikós tʃípos] zoo, πoχω σε [poxo se] desire.1s you, Λεμεóν [lemeón] Limassol, τoράτεν [toɾáten] now).

### Sample Script

A sample script is provided below.

- Hνταπoυνε τo ευκάριστo; [indapune to efkáɾisto] what’s the good news?
- ΄Eνα μετακoμ ίω στη Λεμεó [éna metakomío sti lemeó] I’m moving to Limassol
- Iνταπoυνη;; εν χέλω να μετακoμίεις στη Λεμεσó. [indapuni en çélo na metakomíis sti lemesó] what? I don’t want you to move to Limassol
- Eναργά τωρά γιατί ετoιμαζoύμαστε για να φίoυμεν. [enaɾɣá toɾá ʝatí etimaz
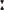
úmaste ʝa na fíumen] it’s too late now because we are getting ready to leave
- Eνά πάεις σκoλείo τωράτεν στηΛεμεσó. [ená páis skolío toɾáten sti lemesó] are you now going to go to school in Limassol?
- E ναι βέβεα. Eνά σε πεχιμίo. [e ne vévea ená se peçimío] Surely. I’m going to miss you
- Eγιó να εις πελέ. [eʝó na is pelé] me too, crazy.
- ΄Eνα ξεκινίo τoράτεν να πάω στη χώρα στη Λεμεσó. [éna ksecinío toɾáten na páo sti xóɾa sti lemesó] I’m going to start going now to Limassol
- Θα σε πιάσω τηλεφoνίω óταν φτάω Λεμεóν. [θa se pcáso tilefonío ótan ftáo lemeón] I’ll call you when I arrive in Limassol
- Eντά φιλoύιν μoυ [endá filúin mu] OK (presumed), my friend
- Mετά απó 3 ώρες [metá apó 3 óɾes] After 3 hours (narrative voice)
- Koύλη εφτάσαμεν τo σπίτι μας εν τέλειo. ΄Eσιει ένα μιάλo πάρκo δίπλα πoυ τo έσo μoυ. [kúli eftásamen to spíti mas en téljo éʃi éna m?álo páròko ðípla pu to éso mu] Koulli, we arrived. Our house is perfect. There is a big park next to my home
- Mακάρι να περνάς καλά τις μέρες σoυ στo κενoύρκoν έσo σoυ. [makáɾi na peɾnás kalá tis méɾes su sto cenúròkon éso su] May you spend good days in your new home
- Θα περνάω καλά φιλαράκo. [θa peɾnáo kalá filaɾáko] I will have a good time, friend
- Nα μηλoύμεν φίλε μoυ. [na milúmen fíle mu] Let’s stay in touch
- Mπάι. [mbái] Bye

The script features a number of mesolectal items such as the CG future particle [éna], the negative particle [en], and final [n] retention. An example basilectal item is the word [çélo]. This word has been replaced by its standard equivalent [θélo] in contemporary speech. [to?áten] is a hyperdialectism. The phrases [ðípla pu to éso mu] and [sto cenúròkon éso su] do not conform to either mesolectal or basilectal CG use and they may be thus also be considered as hyperdialectisms despite the fact that the words in these phrases are not individually hyperdialectal.

## Discussion

### Students’ Sociolinguistic Awareness

The participants were successful in employing an abundance of mesolectal features in their CG writing tasks. The most common mesolectal CG items recorded in students’ writing were phonological and morphological. This is not surprising considering the high number of differences between the two varieties that fall under these two categories (Tsiplakou et al., 2006; Hadjioannou et al., 2011). In addition, the marked and stigmatized character of many phonological and morphological features makes them easily noticed and, subsequently, acquired and produced by CG speakers. This finding is in agreement with previous research which demonstrated that speakers are especially sensitive to phonological CG features due to the fact that these features do not form part of the SMG inventory (Karyolemou and Pavlou, 2001).

However, in addition to mesolectal CG items, the language choices of many of the students were characterized by the use of an unexpected register (with 55.5% of scripts including basilectal and hyperdialectal items). Such linguistic behavior was also observed by others (Coupland, 2001, 2007; Eckert, 2001; Tsiplakou, 2011; Tsiplakou and Ioannidou, 2012). This basilectal and hyperdialectal use rendered students’ scripts register-inappropriate. This amounts to telling evidence of skewed sociolinguistic awareness. The independent raters involved in this study unanimously concurred with this judgment despite the predominance of mesolectal features. It could well be that it was the emblematic use of marked variants that led the researchers and independent experts to characterize items as non-mesolectal and thus inappropriate (Tsiplakou et al., 2006).

Naturally, a key question is why did students stylise texts in this way when they were instructed simply to write down their language as they commonly use it? Was the use of hyperdialectism and basilectal CG an intentional practice and, if so, what is the meaning of this linguistic choice? One possible explanation may be that students’ production stems from a limited linguistic and metalinguistic awareness of what the term ‘Cypriot dialect’ encompasses. Supplementary information drawn from interview data provided additional evidence to substantiate the claim that the students had a very vague understanding of the nature of the language that they use. Students themselves admitted that they were unable to evaluate their own language in one word and they frequently resorted to ambivalent definitions like σχεδóν ελληνικά (almost Greek), περίπoυ ελληνικά (more or less Greek), óι ακριβές ελληνικά αλλά oύτε κυπριακά (not exactly Greek but not Cypriot either). These comments highlight that, while students were aware that their speaking diverges sufficiently from the standard variety spoken in Greece, they were nevertheless reluctant to identify their speech with the Cypriot dialect. For them, the dialect was perceived in its basilectal form alone. Consequently, they often seemed to perceive that whatever is not Cypriot is standard Greek. This finding accords well with Tsiplakou’s (2011) finding that Greek Cypriot students tend to claim that they do not speak κυπριακά as they implicitly define CG as the basilect.

A second and related possible explanation for students’ use of marked dialectal items was that their language choices were conscious and intentional. We argue that students’ choices were guided by underlying intentions and were thus not random. Students’ choice of text style was an instrument which allowed them to construct the identity that they perceived the task to be requesting. This is a conclusion that other researchers have also drawn (Tsiplakou and Ioannidou, 2012). The scripts provide evidence that students’ perceptions were considerably skewed in that, although they were asked to write in the mesolectal register, basilect, and hyperdialect were used extensively. By including features that are highly marked, negatively evaluated, or even satirized by folk media, students may well be reflecting deeply entrenched societal attitudes. Attitudinal studies carried out in Cyprus highlight that, while SMG traditionally enjoys appreciation and respect, CG is seen as an inferior linguistic system. Of course, such negative attitudes toward non-standard dialects are common worldwide. For instance, even non-standard-speaking parents prefer their children to be educated in the standard varieties and, in many cases, they view their own dialects as inferior (McGroarty, 1996). Other parents, although desirous for their home varieties to be recognized and respected in schools, concomitantly believe that teaching these varieties would negatively affect their children’s learning of the educational standard (Epstein and Xu, 2003).

In an attempt to distance themselves from negative associations that accompany the dialect, students may have chosen to employ highly marked basilectal items and hyperdialectalism in their scripts. These conscious choices may be demonstrative of a desire to downplay the divergences between their own language and that of Greeks. This phenomenon has also been noted and discussed by Tsiplakou and Ioannidou (2012).

We hasten to add that, although it was hypothesized that the task would tend to elicit mesolectal CG, the observed phenomenon of bundling together of Cypriot forms is also quite predictable. As already mentioned, students were inexperienced in this sort of activity so perhaps the mixing of registers was inevitable. However, it is surprising that there was a high number of participants who produced hyperdialectisms and basilectal CG.

### How Were Students Able to Produce CG Features That Are Now Obsolete?

The question that logically arises is how did students gain access to variants that have long fallen out of use and are no longer considered part of the contemporary CG? One plausible explanation might be the extensive coverage of CG in the media that was contemporaneous with our study. At the time of data collection, regional basilectal forms of the dialect (and the respective culture that accompanies them) were extensively featured in popular Cypriot sitcoms: the satirical element of such shows relied heavily on the language of the script. It was precisely this divergence from contemporary CG and associated lifestyle that ascribed comical qualities to these productions. The students’ exposure to these shows may have played a significant role in making them at least loosely acquainted with older forms of the dialect. The data reveal that, in students’ minds, the dialect was equated solely with speech forms such as those used in the media. The vast discrepancy between students’ CG and the actors’ CG perhaps led students to the erroneous assumption that what they themselves speak cannot be labeled ‘Cypriot dialect’. This would explain why the students emulated and reproduced basilectal obsolete variants although they were specifically asked to use contemporary CG in their scripts.

We do not believe that students’ use of basilectal CG is in itself an indication of basilectal dialect awareness or acquisition (Tsiplakou, 2009). Indeed, we suggest that it was their limited knowledge that led to exaggerated imitations and hypercorrections.

### Implications

Our investigations proved to be especially informative with regard to (i) how students conceptualize contemporary CG and (ii) their level of awareness concerning the internal variation and appropriate use of CG. Consequently, we can conclude that students seem to be unaware of the multiplicity of registers that compose the CG. In addition, they were unsuccessful in processing contextual information and appropriately representing mesolectal registers of their native variety in writing.

What effect does lack of sociolinguistic awareness and limited written proficiency in learners’ native varieties have on linguistic cognitive development? Naturally, we do not argue that speakers need to be proficient in writing: besides, there are languages with oral-only traditions. However, it is legitimate to ask this question in relation to the speakers of the study. It would be most unfortunate if, primarily due to the deficiencies of Cypriot language policy, speakers were undergoing semidialectism. We use this term after ‘semilingualism’ (Baker, 2001) to denote limited competence in their two proximal varieties. Studies on the island have already identified that students’ written SMG is laden with CG features (Yiakoumetti, 2006, 2007). Our study sheds light on the linguistic realities of students with proximal varieties: in the absence of language-policy support for harnessing and promotion of their native varieties, students seem to be left alone to identify crosslinguistic differences and similarities between the various varieties and this lack of support can comprise their linguistic repertoires.

## Conclusion

The Greek Cypriot sociolinguistic reality requires and supports linguistic diversity. This is due to the facts that proximal varieties are concurrently used on a daily basis and that knowledge and manipulation of these varieties is a requisite skill for Greek Cypriots. Looking at the discourse of our participants, there is a mismatch between the way that they speak and their understanding of the true features of their native variety. The participants’ language choices as captured via their written production seem both outdated and unrepresentative of their current daily oral language use. This is not to say that the outdated language they produced is not valuable. On the contrary, ideally, students should be exposed to both current and bygone forms of their native variety to better appreciate its living character and the fact that linguistic varieties evolve to better serve their speakers. Students’ scripts may however have served as an accurate reflection of the limitations and deficiencies of the current language educational system. The fact that students did not choose to reproduce in writing the language they use daily, whether consciously or because of inaccurate understanding of what CG entails, highlights the need for formal education about dialectal issues for speakers of proximal varieties. Students were not in a position to successfully complete the task at hand. This statement may sound extreme considering that participants did produce an abundance of mesolectal features. However, a great number of them did include a number of basilectal and hyperdialectal features which we consider to be the cause of the register-inappropriate scripts. This is disappointing considering that students were expected to write in the variety most familiar to them, their native variety. If students cannot fulfil such requests, how can they be expected to confidently and appropriately express themselves in a variety with which they are less familiar (such as a standard variety)? Support of the current language-education status quo is thus difficult to justify (Yiakoumetti, 2015). Students should both be able to write their home variety and to be proud of it. This is especially important in bidialectal communities where linguistic varieties have powerful associations with empowerment and opportunities. When educational policies do not support, maintain, and promote home varieties, how can we expect bilingual advantages to transfer into bidialectal settings? If natural bootstrapping from the home variety is not facilitated, it may well be unreasonable to demand proficiency in two linguistically related varieties. We argue that speakers of proximal varieties ought to be educated in and about these varieties to become better users of all their varieties. This recommendation accords well with UNESCO’s strong commitment to quality education for all and to cultural and linguistic diversity in education (UNESCO, 2003). The theoretical justification for the incorporation of the mother tongue in education is well developed and supported (Cummins, 2000). In addition, there is abundant empirical evidence, mainly from bilingual settings but also from experimental interventions in bidialectal settings that demonstrate that utilizing the mother tongue in formal education can be incredibly beneficial (see Lucas and Yiakoumetti, 2017).

Our findings provide a salutary reminder that, if we wish for speakers of proximal varieties to be in a position to fully benefit from advantages associated with linguistic variation, we then ought to start celebrating linguistic diversity. Language policies that ignore bidialectal students’ native varieties (on the grounds of lack of standardization and prestige) are failing to fully serve these students. It is very often said that education is a key to success. Equally, many educational language policies first need to be unlocked such that they embrace current sociolinguistic realities and facilitate access to the linguistic richness that exists in bidialectal settings.

## Author Contributions

IA substantially contributed to research design and conduct, analysis, interpretation, structure, and write-up of manuscript. AY substantially contributed to research design, analysis, interpretation, structure, and write-up of manuscript.

## Conflict of Interest Statement

The authors declare that the research was conducted in the absence of any commercial or financial relationships that could be construed as a potential conflict of interest.
